# Respiratory difficulty caused by an ectopic brain tissue mass in the neck of a two-month-old baby: a case report

**DOI:** 10.1186/1752-1947-5-220

**Published:** 2011-06-08

**Authors:** Mohammed J Aboud

**Affiliations:** 1 Pediatric Surgery Unit, The Maternity and Child Teaching Hospital, Al-Qadisiya, Iraq

## Abstract

**Introduction:**

Neuroglial heterotopia, heterotopic brain tissue, or differentiated neural tissue outside the cranial vault is uncommon, and these anomalies most commonly occur in the nasal cavity.

**Case presentation:**

We report a case of rare pure cystic heterotopic brain tissue in a two-month-old Caucasian baby girl that presented as a large cystic neck mass and was confused with a cystic hygroma. Her mother reported a progressive increase in the size of this swelling and mild respiratory difficulty when the girl was sleeping. A computed tomography scan of the brain and neck showed a large heterogeneous mass extending from the base of the skull to the left submandibular region; a cystic component was also noted. Our patient under went total excision of the cystic mass and prevention of airway obstruction by a left submandibular approach. The final gross pathology diagnosis was heterotopic brain tissue.

**Conclusions:**

Pure cystic neck heterotopic brain tissue lesions are very uncommon, and a preoperative diagnosis of this lesion is difficult. Brain heterotopia is a rare, benign condition that should be considered in the differential diagnosis of the neonatal head and neck mass.

## Introduction

Neuroglial heterotopia, heterotopic brain tissue, or differentiated neural tissue outside the cranial vault is uncommon, and these anomalies most commonly occur in the nasal cavity [1,2]. In rare instances, neuroglial heterotopia in the neck has been reported [3-5]. Previous reports of these cases have shown only computed tomography (CT) images of neuroglial heterotopia presenting as a low-attenuation mass with some area of focal cystic formation [3-6]. The most common location of heterotopic brain tissue is in the nasal region [7,8]. However, heterotopic brain tissue has also less commonly been reported to occur in other sites, such as the pharynx, lung, orbits, palate, tongue, cheek, lip, and neck [9]. To the best of our knowledge, only five or six cases of heterotopic brain tissue with a cystic pattern occurring in the neck have been reported [9-11]. We report a case of uncommon pure cystic heterotopic brain tissue in a two-month-old baby girl that presented as a large cystic neck mass and was confused with a cystic hygroma [12].

## Case presentation

**Figure 1:**
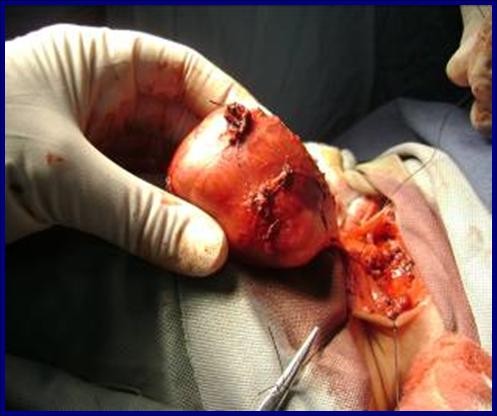
**Peri-operative complete mass excision**.

**Figure 2:**
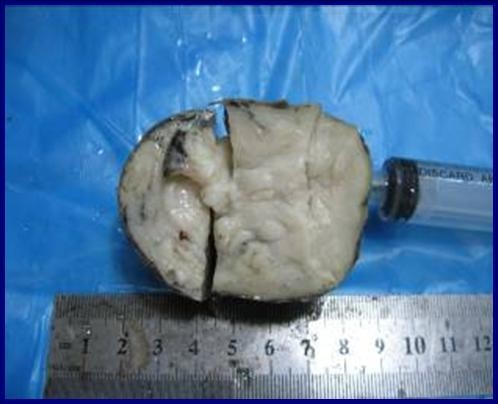
**Gross pathology, showing a grayish mass measuring 6 cm across its largest dimension, with a whitish, soft cut section**.

**Figure 3:**
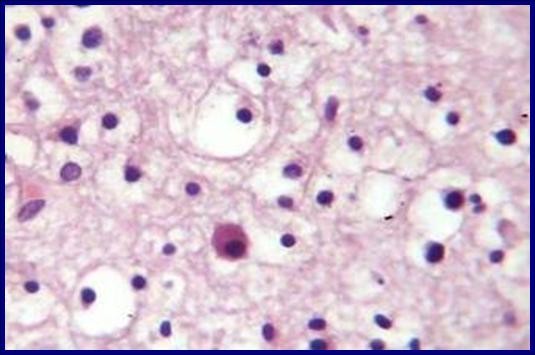
**Histological examination showing neuroglial heterotopia, composed predominantly of glial cells in a neurofibrillary matrix**.

A two-month-old Caucasian baby was admitted to our pediatric surgical ward because of swelling in her left neck region present since birth. She was noted by her mother to have a growing left-sided neck mass and mild respiratory difficulty when sleeping. There was no associated odysphonia or dysphagia. No neck stiffness and no upper respiratory tract infection had been noted in the few days before presentation. Her prenatal history was unremarkable. Our patient experienced snorting, nasal flaring, and an inability to feed on initial evaluation. A physical examination revealed no gross craniofacial abnormalities; our patient was a healthy baby with a large (6 cm × 8 cm) palpable mass in the left neck extending from the upper neck to the supraclavicular fossa. The mass was soft, compressible, non-tender, and fixed. There were no associated changes in the overlying skin. The remainder of the physical examination was normal. A chest radiograph showed a mass lesion in the left neck without extension into the mediastinum. The mass caused mild deformity and deviation of the left mandible and masticator muscles anteriorly. The airway at the pharyngomucosal space was compressed and stenosed. A CT scan of our patient's brain and neck showed a large heterogeneous mass extending from the base of the skull to the left submandibular region; a cystic component was also noted. Findings from a CT scan of the brain were normal. The initial diagnosis was cystic hygroma. Our patient then received total excision of the cystic mass and prevention of airway obstruction by a left submandibular approach (Figure 1). During surgery, a large cystic mass filled with clean fluid over the left parapharyngeal space was noted. The mass adhered strongly to the surrounding tissues. The gross pathology showed a grayish mass, measuring 6 cm across its largest dimension, with a whitish soft cut section (Figure 2). Results of the histological examination showed it to be a neuroglial heterotopia, composed predominantly of glial cells in a neurofibrillary matrix containing a cleft lined by apendymal-like columnar cells and surrounded by meninges, with no malignancy (Figure 3). The final diagnosis was heterotopic brain tissue. There were no post-operative complications and our patient was discharged in good health on the fifth day. No recurrence or complications have been noted in two months of follow-up.

## Discussion

Heterotopic neuroglial tissue (HNT) was first described by Reid in 1852 [13]. Composed of differentiated neuroectodermal tissue, these lesions represent developmental heterotopia of neuroglial tissue rather than true neoplasms [3,10]. Unlike meningoencephaloceles, brain heterotopias lack connection with the subarachnoid space [14,15]. Patients are usually seen initially in the newborn period with airway obstruction, feeding difficulty, or a neck mass. The most common location of HNT is the nasal cavity, where it is traditionally but erroneously termed 'nasal glioma'. Less commonly, brain heterotopias have been reported in the scalp, tongue, pharynx, palate, orbit, and neck [3]. The majority of patients with HNT are products of uncomplicated pregnancies. This anomaly seems to have a left side and female predominance in previously reported cases [3-5], as in our case. Other associated developmental anomalies have been reported, such as cleft palate, Pierre-Robin syndrome, and congenital heart disease [3-5]. The pathogenesis of heterotopic neuroglial tissue is unclear. Several mechanisms have been proposed. CT and MRI scans are complementary studies necessary in pre-operative planning to determine the extent and location of the mass and to exclude intra-cranial connection [16]. Surgical excision is the treatment of choice, although the time it should be performed is controversial. Before surgery, it is difficult to differentiate the neuroglial heterotopia from lymphangioma, as in cases such as our patient. The only clue is a giant single locular (as in our case) rather than multiseptated cyst, which can be commonly seen in lymphangioma; however, heterotopic neuroglial tissue presenting as a giant single locular cyst has been reported on a few occasions. Another way to differentiate these two entities is laboratory study of the fluid.

The treatment for heterotopic brain tissue is complete surgical excision. Surgical intervention is necessary in patients with heterotopic parapharyngeal neuroglial tissue that causes airway distress, dysphagia, or failure to thrive. The timing of surgery is controversial. Proponents of delayed resection believe that resection might be safer in the older child, in whom vital neurovascular structures are more easily salvageable and blood volume is greater [3-6]. Recurrence as a result of incomplete excision has been described [5]. Although focal areas of immature cells have been reported [17,18], these masses lack invasive patterns and are not true neoplasms. Rigorous post-operative care includes attention to nutritional status and speech and swallowing therapy [6].

## Conclusions

In summary, pure cystic neck brain tissue heterotopias are very uncommon lesions, and a pre-operative diagnosis of this lesion is difficult. Heterotopic brain is a rare, benign condition that should be considered in the differential diagnosis of the neonatal head and neck masses.

## Consent

Written informed consent was obtained from the patient for publication of this case report and any accompanying images. A copy of the written consent is available for review by the Editor-in-Chief of this journal.

## Competing interests

The author declares that they have no competing interests.
